# Assessing demand for doctoral-prepared PA faculty: a five-year longitudinal study

**DOI:** 10.1186/s12909-022-03375-x

**Published:** 2022-04-29

**Authors:** Gerald Kayingo, Lucy Kibe, Aldreen Venzon, Karen L. Gordes, James F. Cawley

**Affiliations:** 1 Graduate School, University of Maryland Baltimore, Baltimore, MD USA; 2 College of Science and Health, Charles R. Drew University of Medicine and Science, Los Angeles, CA USA; 3 Betty Irene Moore School of Nursing, University of California, Davis, Sacramento, CA USA

## Abstract

**Purpose:**

Many health profession programs have transitioned to doctoral credentials. While a master’s degree is the terminal degree for the physician assistant (PA) profession, there is increasing discussion regarding the doctoral degree as the PA terminal credential in US higher education.This study examines trends, demand and economic opportunities for doctoral prepared PA faculty; specifically, assessing to what extent PA faculty employers prefer doctoral credentials.

**Methodology:**

This longitudinal retrospective observational study assessed commonly required/preferred academic credentials in PA faculty job postings. Data from 2014 to 2020 was obtained from the labor analytics firm Burning Glass Technologies (BGT) and other academic job search engines. Data on current PA faculty and program directors were obtained from Physician Assistant Education Association (PAEA) survey reports. Wage gap analysis was performed to gain additional insight for the supply and demand of PA educators with a doctoral degree.

**Results:**

Of the 612 unique job ads posted from 232 PA programs between 2014 and 2020, approximately 38.9% (238) stated a preference or requirement for a doctoral degree. Nearly half of the postings for program directors and leadership positions preferred candidates with doctorates. There was a correlation between tenure eligibility positions (20.1%) and preference/requirement for doctoral credentials. PAEA survey data (2014–2019) revealed approximately 24% PA faculty and 45–48% of program directors held a doctoral degree with Doctor of Philosophy (PhD) as the most frequently held doctorate. No significant difference existed in wages for faculty with or without doctoral degree.

**Conclusions:**

Based on a national sample of PA program job ads, there is a preference for doctoral-prepared PA educators and the demand for these candidates is greater than market supply. Our analysis has implications for individual faculty career planning, employers and the PA profession as it debates transition to a terminal doctoral credential. Further studies should assess the impact of doctoral credentials on PA education by examining measurable outcomes.

## Introduction

During the past two decades, there has been expansive growth and evolution in the field of PA education [1]. Following the 2009 determination by the profession that the master’s degree was the terminal degree awarded for entry-level PA professional education [2], the number of PA programs continued to rise rapidly, and discussion of doctoral degrees was largely muted. More recently, however, there has been the emergence and growth of post-professional doctoral (PPD) degrees [3]. In 2020, a formal exploration on adopting the doctoral degree as the entry-level degree for PAs was conducted by the Physician Assistant Education Association (PAEA). After deliberation, members voted against adopting the entry-level PA doctorate, but promoted advancement of academic pursuit, including doctoral degrees in other fields of study. Nonetheless, our analysis, which included assessments of marketplace demand and returns on investment, suggests that there is increasing demand for opportunities for doctoral-prepared PA faculty.

Within the PA profession, the number of certified PAs with a doctoral degree has increased from 1,893 (1.7%) in 2016 to 2,604 (2.0%) by 2020 [4]. These proportions have been slowly increasing in the past five years as only 1.2% of all certified PAs had a professional doctorate in 2014 [5]. Among PA educators, 208 (23.9%) of faculty and 58 (43.6%) of program directors reported that their highest completed degree was a doctoral degree in 2017. These numbers had increased to 222 (23.5%) faculty and 95 (45.5%) program directors by 2019 [6–9]. In related fields such as nursing, a majority of faculty have doctoral credentials with 88.6% of vacant faculty positions requiring or preferring a doctoral degree [10]. The Institute of Medicine has recommended doubling the number of doctorally prepared nurses in the United States by 2020 to ensure that sufficient numbers of faculty are available to prepare the nursing labor force that is needed for delivery of healthcare services [11].

There exists at present a shortage of information on the current demand for and opportunities available to PA educators who hold doctoral degrees. In addition, current expectations for PA faculty are increasingly changing to include not only teaching but also scholarship, leadership, service, and working with students in other professions to meet the requirements of interprofessional education. This study aims to investigate current trends, demand and opportunities for doctoral prepared PA faculty. What are some of the drivers of the academic marketplace for PA faculty with doctoral degrees? There were two specific study purposes: 1) To characterize current trends among PA faculty with doctoral degrees, and 2) To assess the marketplace demand and academic opportunities available to doctoral prepared PA faculty. To gain additional insights on whether unfavorable return on investment was a factor in assessing the supply of PA educators with a doctoral degree, a wage gap analysis estimating net present values (NPV) under several scenarios was also performed.

## Methods

We conducted a retrospective, longitudinal quantitative analysis of 1) online job postings of PA faculty that prefer or require a doctoral degree and 2) trends in the number of PA faculty who hold a doctorate degree and type of degree held over a 5-year time period. The former was used as an indication of demand for doctoral preparedness. To our knowledge, this is the first study to elucidate the demand of a doctoral prepared PA faculty-using job posting data. Additional analysis was performed on other attributes of the online job advertisements including tenure eligibility, leadership experience and potential regional variation.

Job posting data for 2014 was obtained from one of the leading labor analytics firms Burning Glass Technologies (BGT) [12, 13] and data for 2015–2020 was obtained from academic job search engines that included chroniclesvitae.com, HigherEDJobs, PAEA JOBS, monster.com, Indeed, CareerBuilder, Simply Hired, LinkedIn and glassdoor.com. Job postings that were included in this study (inclusion criteria) were academic job postings for physician assistants (PA) and contained a job description, educational degree requirement or preference, and were located within the United States regions and divisions. Exclusion criteria included clinical job postings for PAs, those without a job description, were outside of the United States or was a duplicate job posting. After sorting the 2014–2020 job posting data to avoid duplications, we analyzed 612 unique PA faculty job ads from 232 PA programs of which 142 (23.2%) postings were for program directors, chairs or higher and 470 (76.8%) postings were for principal and adjunct faculty (grouped as regular faculty for this study). These PA faculty job postings covered 232 PA programs from 45 states, the District of Columbia and the territory of Puerto Rico. The states not represented in these job ads were Alaska, Delaware, Hawaii, Idaho and Wyoming. To investigate the actual trends of current faculty who have a doctoral credential, we analyzed data from the Physician Assistant Education Association (PAEA) national PA Program Faculty and Directors Survey reports covering the period from 2014–2019. Data on current PA faculty and program directors were obtained from the Physician Assistant Education Association, Faculty & Directors Survey and Reports 2014, 2015, 2017, and 2019. Data on the average salary of certified PAs was obtained from the National Commission on Certification of Physician Assistants.

Prior to data analysis, the job posting text and title were cleaned and processed using different programming languages, including Python version 3.6.5. Key variables of interest included job description text, title, faculty role, advertising institution, state/ region in the USA, required degree and preferred degree, minimum requirements, tenure eligibility. The required and preferred educational degrees were categorical variables such as masters or doctorate. Positions requiring and those preferring a doctorate were lumped in one category. Data were analyzed descriptively. We focused on proportions of job postings rather than absolute numbers. To evaluate how job market demand compares with existing supply, PA faculty job postings were compared with PAEA data on faculty and program directors who currently hold a doctoral degree.

To gain insights into the projected return on investment of a PA with a doctorate at retirement age we estimated the sum of future cash flows of PAs in clinical practice and those in academia for 40 annual payments assuming a 10% annual salary growth rate and a 5% discount rate. Salaries for PA educators and clinically practicing PAs were obtained from PAEA [9] and NCCPA [4] data respectively. The sum of future cash flows was calculated using the formula below, where ***P*** is the current median salary, ***g*** is the assumed salary growth rate (10%), ***r*** is the assumed discount rate and ***n*** is the number of years of employment (40 years). Salary excludes fringe benefits and includes only income from a principal clinical position (part-time or moonlight positions not included). Income for faculty working at 0.75 FTE or greater, wages are not adjusted for years in practice, gender, race or rank). Calculation on sum of future cash flows did not take into account time lost due to additional school to obtain a doctoral degree. Taxation was also not factored into the calculation. The discount rate is the rate of financial return used in a discounted earning analysis to determine the present value of future earnings. Although various researchers have used different rates for discounting, a figure of about 5% is a reasonable estimate. It is between the average historical returns in the US stock market (8%) and the long-term return of on US Treasury securities (2%). A discount of 5% was also chosen for this study to be consistent with previous studies that have analyzed the value of education as an investment. Statistical analysis was conducted using SPSS to assess whether wage differences observed across various groups were significant.

$$PV=\frac{P}{1+r}+\frac{P\left(1+g\right)}{{(1+r)}^{2}}+\cdots +\frac{{P(1+g)}^{n-1}}{{(1+r)}^{n}}$$

## Results

Of the 612 unique job ads that were posted from 232 PA programs between 2014 and 2020, approximately 38.9% (238) stated that they preferred or required a candidate with a doctoral degree. (Table 1). Approximately 50% of chair and/or program director job ads preferred or required a doctoral degree. Of the 470 regular faculty positions analyzed, 167 (35.5%) preferred or required a doctoral degree, reflecting about one in every three faculty job postings. Out of 612 postings, 123 (20.1%) indicated that the position was eligible for tenure. Seventy-seven (62.6%) of the 123 positions that offered tenure, preferred or required a doctoral credential. Therefore, both tenure and higher leadership positions were more likely to prefer doctoral candidates. For all leadership faculty jobs with a dean title, doctoral degree was the required minimum credential (data not shown). For all other faculty jobs, a master’s degree was the required minimum credential (data not shown). When we compared states and types of universities, we found no significant difference between various regions of the US or between liberal arts colleges’ vs academic medical centers or between public and private institutions concerning the doctoral credential preference for jobs ads between 2015–2020. However, for the job ads for 2014, the southern region of US had the most job posting that preferred a doctoral degree at 62 (44.0%), while the western region had the least at 14 (9.9%) out of all the preferred doctoral job postings in 2014.

**Table 1:** PA Faculty Job Ads (2014–2020)^a^

PA Faculty Job Ads (2014–2020)	Count	Percentage (%)
Total number of unique job ads analyzed	612	100
Program Director/ Chair/Division Chief Job Ads	142	23.2
Principal faculty/adjuncts/research director job ads	470	76.8
Tenure track/eligible positions	123	20.1
Total positions that required or preferred a doctoral credential	238	38.9
Program director job ads that required or preferred a doctoral credential	71	50.0
Principal faculty job ads that required or prefer a doctoral credential	167	35.5
Tenure track/eligible job ads that required or preferred a doctorate	77	62.6

^a^Data for 2014 are from Burning Glass Technologies and for 2015–2020 are from other academic job search engines

**Fig. 1:**
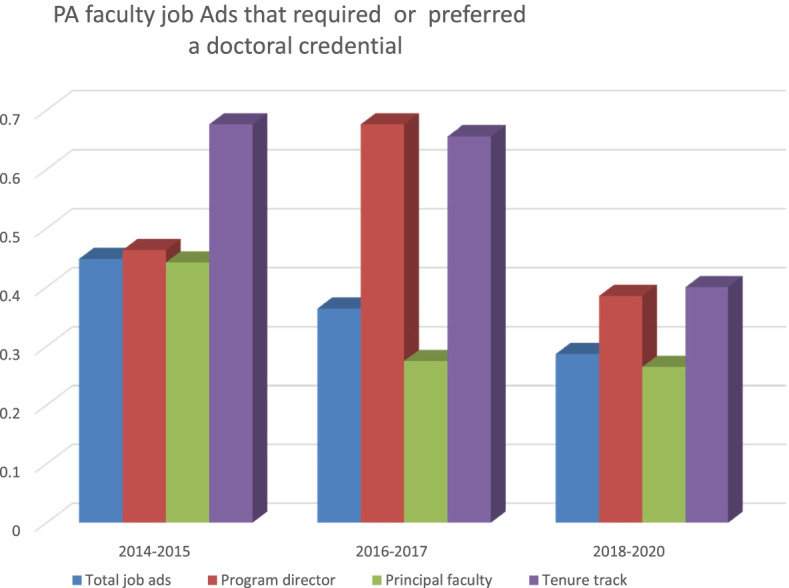
PA faculty positions requiring/preferring doctoral degrees*

Trend analyses of job ads that preferred or required a doctoral degree for the years between 2014 and 2020 are shown in Fig. 1 and Table 2. While the overall numbers of ads requiring or preferring a doctoral degree increased over time, the proportions of those preferring/requiring a doctoral degree compared to the total job ads posted has been declining. This is probably explained best by exponential growth of the PA profession and the sheer increase in the number of PA programs over this time period. By 2014, over 40% of all job postings preferred candidates who are doctorally prepared compared to approximately 29% by 2020. Similarly, the proportion of program director ads that preferred a doctoral degree declined from 46.3% in 2014 to 38.5% by 2020.

**Table 2:** PA faculty job ads requiring/preferring doctoral degrees*

% of job ads that require or prefer candidates with a doctoral credential				
*Year*	***Total job ads***	***Program directors***	***Principal faculty***	***Tenure track/eligible jobs ads***
2014–2015	44.80%	46.30%	44.20%	67.60%
2016–2017	36.36%	67.60%	27.50%	65.60%
2018–2020	28.70%	38.50%	26.50%	40%

### Trends among doctoral-prepared PA faculty

**Fig. 2:**
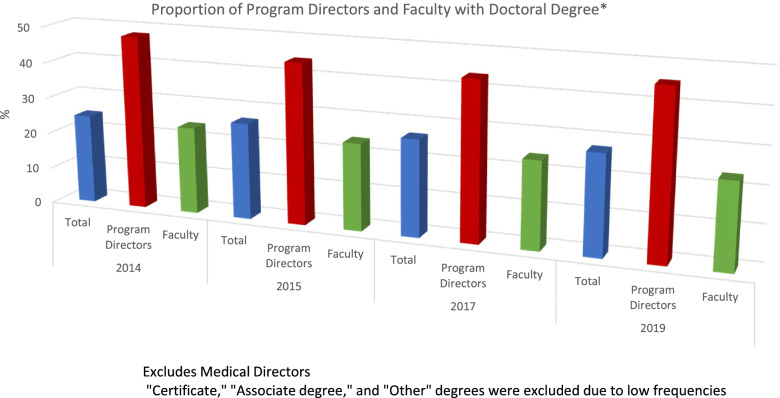
Proportion of program directors and faculty with doctoral degrees. Between 2014 and 2020

To gain more insight into the demand and supply of PA faculty with a doctoral degree, we investigated the actual trends of current faculty who have doctoral credentials using the national PAEA Faculty and Directors reports [6–9]. Although the number of doctoral degree holders increased between 2014 and 2019, the proportion of PA faculty and program directors with doctoral degrees remained steady with a combined statistic of 27%. During this period, approximately 24% PA faculty and 45–48% of program directors reported having a doctoral degree (Fig. 2). Regarding tenure status, the proportion of faculty who reported having tenure or being on tenure track has remained flat at about 20% for the past five years (data not shown).

**Fig. 3:**
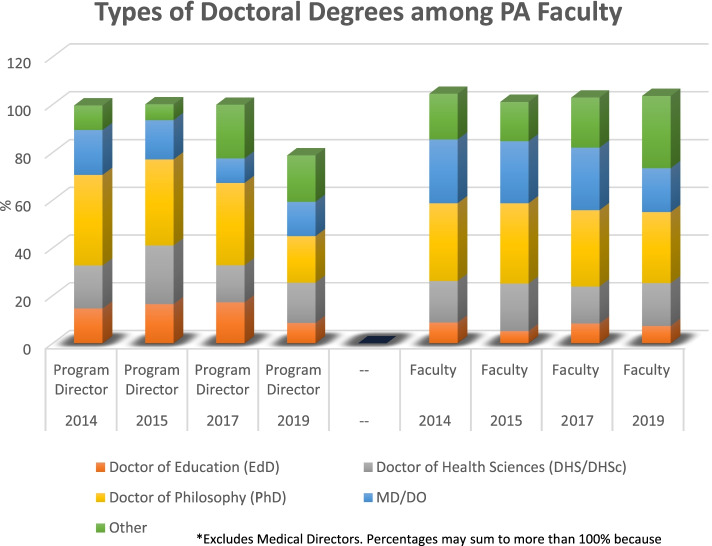
Types of doctoral degrees among PA faculty*

Doctoral degrees were reported in more than 10 areas of study. Figure 3 shows the distribution and areas of study commonly held by PA faculty and program directors over time. The Doctor of Philosophy (PhD) was the most frequently held doctoral credential for both faculty and program directors across all years investigated but declined in 2019, especially among Program Directors. For instance, the proportion of PhD holding faculty declined from 32.6% (*n* = 56) in 2014 to 29.8% (*n* = 65) in 2019 and from 38% (*n* = 19) in 2014 to 19.5% (*n* = 23) in 2019 for program directors. In general, the Doctor of Health Science (DHS/DHSc) is the second most common degree and has remained fairly stable and similar between program directors (range: 15.5–24.6%) and faculty (range: 15.5%-19.9%). The Doctor of Education (EdD) comes third. The EdD credential is more popular among program directors compared to faculty but declined from 14.6% in 2014 to 8.5% in 2019. Among faculty, the EdD remained stable (range: 5.1%-8.7%). Among MDs or DOs who are program directors or regular faculty, but not medical directors, we observed a downward trend over time (program directors: 18.8% in 2014 and 14.4% in 2019; faculty: 26.7% in 2014 and 18.4% in 2019). The remaining doctoral areas of study were combined into an ‘other’ category as shown in Fig. 3. Of note, Doctor of Medical Science (DMSc), an emerging post-professional doctoral degree among PAs, was reported as a distinct area of study in 2019. Only 2.3% (*n* = 5) of faculty and 3.4% (*n* = 4) of program directors were reported holding this degree. Overall**,** a comparison of job ads and the status of already employed PA faculty may indicate that there is not a large pool of doctoral PA candidates the market can supply. Hiring academic institutions may be settling for non-doctoral PA faculty candidates because this is what they can readily get in the labor market even if their job ads specify a preference for candidates holding doctoral credentials. This observation correlates with our personal experiences, anecdotal reports from hiring deans and departmental chairs. Data collected in 2019 by PAEA (before the COVID pandemic) shows that there were about 114 vacant faculty positions. These vacant PA faculty FTES were reported by about 21% (87 programs) across all reporting programs the US. The PAEA report also reveals that 196 (83.4%) programs reported seeking to hire new faculty or staff in the 2018–2019 academic year, before the COVID-19 pandemic [9] further highlighting the demand for PA faculty in general. However, the results on tenure expectations among job ads (20.1%) are consistent with the current tenure status of PA educators as reported in the PAEA national surveys of PA Program faculty and directors over time [6–9].

### Wage gap analysis

A wage gap analysis was performed to gain more insights on whether unfavorable return on investment could be a factor into the limited supply of PA educators with a doctoral degree. (Table 3). Salaries for PA educators and clinically practicing PAs were obtained from PAEA [9] and NCCPA [4] data respectively. The mean and median income of a certified PA in clinical practice were higher than that of certified PA in academia by $13,367 and$ 8,000 respectively. Academic PAs with a doctoral degree also make less money than clinically practicing PAs without a doctoral degree. The salary difference between a regular PA-faculty with doctoral degree and a regular faculty without a doctoral degree was very small. In contrast, program directors with a doctoral degree receive significantly higher salaries (> 20%) compared to PAs in clinical practice or regular PA faculty without a doctoral degree (*p* < 0.0001). Projected cash flows of a regular PA faculty with a doctorate at retirement age were higher than those of regular faculty without a doctoral degree but lower than those PAs in clinical practice. When one factors in at least one year of lost income due to additional school, and student debt incurred when getting a doctoral degree, the Net Present Value (NPV) of a regular PA faculty with a doctorate is lower than that of a regular faculty without a doctorate and that of a PA in clinical practice. The 40 year estimated cash flow for program directors with a doctoral degree ($15,200,869) is the highest of all categories of PA employment analyzed. Even when investment costs such as student debt, interest and lost revenue due to additional schooling are considered, the positive NPV associated with a doctoral degree among program directors is higher than those of other PA roles at retirement age of 65 years.

**Table 3:** Wage gap analysis: comparing annual base faculty salary with income of a certified PA in clinical practice

PA position	Mean	Median	∑of future cash flows
Certified PA in clinical practice	$113,186	$105,000	$11,400,652
Certified PA-faculty	$99,819	$97,000	$10,532,031
Certified PA-Program director	$134,306	$131,000	$14,223,670
PA-faculty with Doctoral degree	$106,203	$103,000	$11,183,497
PA-Program Director with Doctoral degree	$142,215	$140,000	$15,200,869
Wage Gap between clinical and academic PA	($13,367)	($8,000)	($868,621)
Gap between clinical & academic PA + a doctoral degree	($6983)	($2,000)	($217,155)

## Discussion

This study examined current trends, demand and economic opportunities for doctoral prepared PA faculty; specifically, assessing to what extent PA faculty employers prefer doctoral credentials. The main findings were that about one in every three postings preferred candidates with a doctoral degree, and nearly half of the postings for program directors and majority of tenure and higher leadership positions preferred candidates with doctorates.

What is driving the demand for PA faculty with doctorates? Over the past two decades, PA faculty have become integral members of academic departments within universities, subject to the same merit and promotion policies as faculty in comparable health professions. To be successful in academe, a doctoral credential seems to be necessary for success in the long run. Academic employers are seeking candidates with credentials that will ensure parity with other faculty in a given department. Given the continuing expansion of the number of PA programs (now at 282 accredited programs) [14] there is a need for more doctoral faculty to meet this growing demand. Additionally, the expectations for PA faculty have also changed over time to include scholarship, inter-professional education, service and leadership at the local and national level. A doctoral credential would likely open doors for PA faculty in such roles. Importantly, attaining tenure and higher leadership positions are more likely to require doctoral candidates.

In the present study, of all doctoral degree credentials held, the PhD was the most common, and generally most highly regarded by academic institutions for promotion and tenure success. Most clinical doctorates aimed at PAs do not emphasize research as compared to traditional academic doctorates. Moreover, newer clinical doctorates such as the DMSc may be unfamiliar to institutional deans and promotion committees. The current PA master’s degree curriculum is largely clinical with a principal goal of producing practicing clinicians who will enter the workforce with a primary role of providing patient care. Employers in academic settings may be looking for additional graduate training to ensure the PA professoriate has adequate training in pedagogy and has developed competencies to succeed in a faculty role. PhD programs in education or health professions education may be better equipped for this market.

It is also interesting to note that while the percentage of PA educators holding doctoral degrees has increased in the past decade, levels of scholarship have dropped over the same period. The mean number of total publications was 2.7 (down from 4.2 in 2010); the median was zero with 50.6% reporting no publications during their career, a finding that was attributable to the recent influx of junior faculty [15].

Another factor driving the demand for PA faculty with doctoral degrees is that PA programs have transitioned to graduate level education, a requirement to be accredited by the ARC-PA [14]. The percentage of master’s degrees held by certified PAs has increased from 66.2% in 2013 to 77.2% in 2019. As noted, in 2020, there have been discussions to transition the PA terminal degree from a masters to a doctorate. Anticipating these changes, it makes sense for employers to think ahead and hire faculty who have the doctoral credential.

Comparison of the proportion of job ads preferring or requiring a doctorate with the proportion of current faculty (already hired) who already have a doctorate suggest a high labor market demand for PA educators with a doctoral degree. The limited supply could be due to the fact that most PAs are educated primarily as clinicians where a master’s level terminal degree is sufficient. Other possible causes of the unmet demand may include aging of faculty, retirements and low pool of PA educators being produced. The lack of opportunities for advancement and the uncertainties about return on investment for advanced degrees could also deter PAs from acquiring doctoral credentials [16, 17]. Our wage gap, cash flow and present value analysis suggest that there may not be a favorable return on investment to obtaining additional degrees among clinical PAs beyond the current terminal master’s degree. Benefit is observed for PA educators in leadership positions such as program directors and/or departmental chairs. To mitigate possible concerns about return on investment, employers of PA faculty could provide incentives and protected time for master level trained faculty to advance to doctoral education. Initiatives such as grow-your-own [18] could also increase the pool of PA faculty with doctoral degrees. Alternatively, the PA profession may consider admitting second career candidates who already have doctoral education prior to becoming PAs [19].

Health professions education is changing around the globe with experts calling for reforms to accelerate the pool of competent and advanced trained educators who will transform education, strengthen health systems and advance health [20, 21]. In nursing for instance, the number of faculty with a PhD in nursing increased by 921 (19%) from 2008–2017 while the number of faculty with a DNP increased by 2,022 (123%) during the same period [22]. This study adopted a unique methodology of using job ads to investigate current trends, demand and opportunities for doctoral prepared PA faculty. The data sets used were comprehensive and expected to reflect well over 80% of actual PA job openings [12, 13, 23]. Study limitations include self-reported data in the national PA faculty and program surveys. Our income gap analysis doesn’t consider various scenarios such as promotions and does not control for several potential confounders such as length of employment, gender, or race.

In summary, our findings indicate that there is a greater demand for PA faculty with doctoral degrees than currently exists in the market supply. To help meet this demand, some universities have created post-professional PA degrees such as the doctor of medical science (DMSc). Whether or not the rigor of these programs will yield the outcomes required by educational institutions has yet to be determined. There is also appears to be long-term benefit to PA educational programs to continue to preferentially hire doctorally-prepared faculty in terms of increased probability of promotion and attaining tenure, advancing administratively, and greater financial return on investment. In depth investigations will be necessary to further elucidate the drivers of the academic marketplace for PA faculty with doctoral degrees. Also further studies are needed to address which type of doctoral education is most relevant for PAs in academia and to evaluate the impact of doctoral credentials on PA education, practice and return on investment.

## Data Availability

Job analysis data for 2014 retrieved from Burning Glass Technologies with permission, job analysis data for 2015–2020 was retrieved from academic job search engines The datasets used and/or analyzed during the current study are available from the corresponding author on reasonable request.

## Abbreviations

AAPA: American Academy of Physician Assistants
DO: Doctor of Osteopathic Medicine
MD: Doctor of Medicine
DMSc: Doctor of medical science
NPV: Net Present Values
OTP: Optimal Team Practice
PA: Physician Assistant
PAEA: Physician Assistant Education Association
PPD: Post-professional Doctoral Degree
